# A Move in the Right Direction

**Published:** 1887-11

**Authors:** 


### A Move in the Right Direction.

The announcement recently made by the College of Physicians and Surgeons of New York to the effect that, beginning with the session of 1888–9, and thereafter, it will require preliminary examination of matriculants who do not hold diplomas or certificates from recognized colleges or schools of science, should be hailed with satisfaction by the profession.

If anything is needed at the present time it is a reform in our present system of medical education. Under the exercise of old custom students of any and every grade of preliminary training have been freely admitted to courses of medical instruction by nearly every medical school in this country. With absolutely no safeguardes thrown around the study of medicine, it is not a matter of surprise that the ranks of the profession of medicine are filled with ignorant, illiterate, and incompetent men. The extent of this ignorance and illiteracy can only be appreciated by those who have the means of coming into contact with large numbers of medical practitioners. To the medical editor these revelations of incompetency are frequent and appalling. We are frequently in receipt of communications from medical practitioners which display such illiteracy and ignorance that we can not but regret that their prior general training was so utterly deficient as to defeat their usefulness in professional work to a very great extent. Whilst we admit that an occasional man may make an excellent practitioner with a very small knowledge of the language he speaks, this argument will only hold good in such exceptional cases as make it a dangerous precedent. Other things being equal the better preliminary education the medical student has the better he is equipped for the study and practice of his profession. He should at least have a good knowledge of his own language and some acquaintance with Latin and Greek, but especially the former. He should know something of elementary mathematics, including arithmetic, algebra, and plain geometry, and should have some acquaintance with general history and modern literature. Those young men who have not enjoyed the advantages of preliminary instruction to the extent here indicated should be discouraged by their preceptors from studying medicine and should be refused admission to our medical schools until they have passed a satisfactory examination on these branches.

The action of the College of Physicians and Surgeons of New York in requiring a preliminary examination is a move in the right direction and we trust that their example will be the means of inducing other schools to adopt this course.—*Maryland Medical Journal*, August 13, 1887.

